# Evaluation of Asthma/Atopic Outcomes in Relation to Breastfeeding Status Among HIV-Exposed Uninfected and HIV-Unexposed Uninfected Children

**DOI:** 10.3390/pediatric18050130

**Published:** 2026-09-29

**Authors:** Tamar A. Smith-Norowitz, Samantha Yanez Perez, Sabah N. Islam, Rauno Joks, Stephan Kohlhoff

**Affiliations:** 1 Department of Pediatrics, SUNY Downstate Health Sciences University, Brooklyn, NY 11203, USA; samantha.yanezperez@downstate.edu (S.Y.P.); sabah.islam@downstate.edu (S.N.I.); stephan.kohlhoff@downstate.edu (S.K.); 2 Department of Pathology, SUNY Downstate Health Sciences University, Brooklyn, NY 11203, USA; 3 Department of Medicine, SUNY Downstate Health Sciences University, Brooklyn, NY 11203, USA; rauno.joks@downstate.edu

**Keywords:** HIV-exposed uninfected children, atopy, breastfeeding

## Abstract

Objectives: Perinatal Human Immunodeficiency Virus (HIV) exposure may contribute to the development of asthma and atopic diseases through alterations in immune regulation. While an increased prevalence of atopic conditions has been reported among perinatally HIV-infected children, less is known about children who are HIV-exposed but uninfected (HEU). Breastfeeding provides important benefits for infants; until recently, breastfeeding was not recommended in women living with HIV (WLHIV). The focus of this study was the evaluation of asthma/atopic outcomes in relation to breastfeeding history among HEU and HIV-unexposed uninfected (HUU) children. Methods: A retrospective chart review (2005–2026) that examined pediatric medical records of children (M/F, 0–21 y/o) at an academic medical center (Brooklyn, NY) who were either perinatally HEU (N = 67) or HUU (N = 58). Data collected included infant-feeding practices (breast milk exposed or not exposed), demographics, medical and family history, and physician-diagnosed asthma or atopic conditions. Results: A medical diagnosis of atopy or asthma was associated with perinatal HIV exposure across both groups (*p* = 0.006) (Fisher exact test). HEU and HUU children were either breastfed (N = 13, 54, respectively) or not (N = 54, 4, respectively). However, when comparing exposure to breast milk versus no breast milk exposure, there was no association between perinatal HIV exposure and asthma or atopy in either group (*p* = 0.31, 0.19, respectively). Conclusions: Among both breast-milk-exposed and non-exposed children, there was no statistically significant difference in asthma/atopy prevalence between HEU and HUU children.

## 1. Introduction

Advances in the use of antiretroviral therapy (ART) for the prevention of mother-to-child transmission of human immunodeficiency virus (HIV) have resulted in a growing population of children who are HIV-exposed but uninfected (HEU) [1]. Although HEU children do not acquire HIV infection, studies suggest that perinatal HIV exposure may alter immune development and increase the risk of adverse health outcomes compared with HIV-unexposed uninfected (HUU) children [2,3]. These observations have raised concerns that perinatal exposure to maternal HIV infection, chronic immune activation, and antiretroviral medications may have lasting effects on immune function beyond infancy.

Immune dysregulation during early life has been proposed as one mechanism that may alter health outcomes among HEU children [2,3]. Maternal HIV infection is associated with systemic inflammation and changes in the fetal immune environment that may influence neonatal immune maturation. Studies have demonstrated differences in T-cell function, cytokine production, innate immune responses, or monocyte activation in HEU compared with HUU infants, suggesting that perinatal HIV exposure may have future immunologic consequences [2,3]. Increased prevalence of asthma and atopy has been shown among children with perinatally acquired HIV infection; however, data on HEU children remain limited [4].

Breastfeeding influences infant immune development and is associated with childhood asthma and atopy, although findings have been inconsistent [5,6]. Historically, women living with HIV (WLHIV) in the United States (U.S.) were advised not to breastfeed, making it difficult to distinguish the effects of perinatal HIV exposure from those of infant-feeding practices; however, more recently, guidelines have endorsed feeding of breast milk as a possible option [7].

While previous investigations have identified an association between perinatal HIV exposure and increased risk of atopic disease [8], the contribution of breastfeeding practices to this risk has not been well characterized. This study involved a secondary analysis of data originally collected as part of the study described by Islam et al., which examined the long-term health effects of perinatal HIV exposure [8]. The current analysis addresses a distinct question that was not examined in the original study: evaluating asthma/atopic outcomes in relation to breastfeeding history among HEU and HUU children. These analyses are exploratory comparisons of disease prevalence by HIV exposure status and feeding among HEU and HUU children.

## 2. Materials and Methods

### 2.1. Data Source and Sample Selection

We performed a retrospective cohort study using encounters from electronic health records (EHRs), including HealthBridge (Cincinnati, OH, USA), Office Practicum (Fort Washington, PA, USA), and EPIC (Epic Systems; Verona, WI, USA) for pediatric patients receiving care at an academic medical center in Brooklyn, New York, between 2005 and 2026.

### 2.2. Study Population

Medical records of children and adolescents aged 0–21 years were reviewed based on the charts from our original study of Islam et al. [8]. (Patients were added for the year 2026.) The participants were categorized into two groups based on perinatal HIV exposure status: HIV-exposed but uninfected (HEU; N = 67) and HIV-unexposed, uninfected (HUU; N = 58). HUU were identified from the same clinical setting as HEU participants. Patients were identified through a retrospective search of the EHR using predefined diagnostic codes and relevant clinical criteria during the study period based on studies of Islam et al. [8]. The search was designed to identify all potentially eligible patients with perinatal HIV exposure and HIV-unexposed comparison patients who met the study eligibility criteria [8]. If multiple records were available for the same patient, the records were consolidated into a single patient-level dataset.

Data extracted from the health record system included demographic characteristics, infant-feeding practices (breast milk exposed or not exposed), medical history, family history, and physician-documented diagnoses of asthma and/or atopic diseases. Atopic diseases included clinically diagnosed allergic conditions documented in the medical record (i.e., atopic dermatitis, allergic rhinitis). Asthma, allergic rhinitis/conjunctivitis, atopic dermatitis, and IgE-mediated food allergy were analyzed both individually and, where applicable, as a composite asthma/atopic disease outcome.

### 2.3. Feeding Groups

Children in the HEU and HUU breastfed group were exposed to breast milk versus no breast milk exposure.

### 2.4. Variable Definitions. Outcomes

Studies in our laboratory by Islam et al. examined the long-term health effects of perinatal HIV exposure [8] and the prevalence of physician-diagnosed asthma and/or atopic disease. In the current study, associations between perinatal HIV exposure and the presence of asthma or atopy were re-evaluated and expanded upon. Markers of atopy and/or asthma included review of medication prescriptions and a physician-made diagnosis of moderate to severe asthma, allergic dermatitis, or AR/conjunctivitis, as well as IgE-mediated food allergies as documented in the medical record. History of the mother’s HIV status, family history of atopy and/or asthma diagnosis was collected. Sociodemographic characteristics analyzed included age, gender, race and ethnicity, asthma, or atopy.

The secondary analysis focused on the evaluation of asthma and atopic outcomes in relation to breastfeeding history among HEU and HUU children.

### 2.5. Statistical Analysis

We performed statistical analyses using SAS software v9.4.3 (SAS Institute Inc., Cary, NC, USA). A two-sided *p*-value < 0.05 was considered statistically significant.

### 2.6. Ethics Approval

This study was reviewed by the SUNY Downstate Health Science University Institutional Review Board (IRB) and approved as non-clinical intervention and exempt research (Protocol # 307753-1). Patient consent was waived because of the retrospective design. The procedures followed were in accordance with the ethical standards on human experimentation and with the Helsinki Declaration of 1975 (revised in 1983).

## 3. Results

### 3.1. Study Cohort

A total of 125 children were included in the analysis, including 67 children who were HEU (mean age 5 ± 5 years) and 58 children who were HUU (mean age 4 ± 4 years). Demographics and characteristics of the cohorts are listed in Table 1.

### 3.2. Association Between HIV Exposure Status and Asthma/Atopy According to Feeding Status

In the overall study population, asthma/atopy was documented in 39 of 67 (58.2%) HEU children compared with 19 of 58 (32.8%) HUU children (OR, 2.86; 95% CI, 1.29–6.33; Fisher’s exact *p* = 0.006). Among children who were exposed to breast milk, asthma/atopy was documented in 6 of 13 (46.2%) HEU children and 18 of 54 (33.3%) HUU children (OR, 1.71; 95% CI, 0.54–5.46; Fisher’s exact *p* = 0.31). Among children who were not exposed to breast milk, asthma/atopy was documented in 33 of 54 (61.1%) HEU children and 1 of 4 (25.0%) HUU children (OR, 4.71; 95% CI, 0.46–48.43; Fisher’s exact *p* = 0.19). These breastfeeding-stratified analyses should be interpreted cautiously because of the small number of HUU children in the not exposed to breastmilk subgroup and the marked imbalance in breastfeeding status between the HEU and HUU groups. A statistically significant association was not demonstrated within either breastfeeding stratum (Table 2).

## 4. Discussion

In the current study, there was no difference in the prevalence of asthma or atopy between children who were exposed to breast milk and those who were not, among HEU and HUU. Prior literature reported that HEU children exhibit persistent alterations in immune development, including differences in inflammatory responses, T-cell function, and susceptibility to immune-mediated conditions despite the absence of HIV infection [1,9,10]. The mechanisms underlying these immune responses remain unclear, but may involve maternal immune activation, in utero exposure to antiretroviral therapy, changes in microbial colonization, or differences in early-life immune maturation [1,11].

This is an exploratory study that compared disease prevalence according to HIV exposure and feeding status. Breastfeeding provides important immunologic benefits through the transfer of maternal antibodies, cytokines, immune cells, human milk oligosaccharides, and other components that influence infant immune development and intestinal microbiome composition [12,13]. However, the relationship between breastfeeding and allergic disease outcomes remains complex; studies of the general population demonstrate inconsistent associations between breastfeeding and subsequent asthma or atopy [14]. Regarding maternal HIV infection, breastfeeding practices have historically been restricted due to concerns of postnatal HIV transmission. However, current guidelines now support breastfeeding for selected WLHIV who are receiving antiretroviral therapy and maintaining viral suppression [15]. Although the risk of HIV transmission during breastfeeding is estimated to be less than 1% with sustained viral suppression [7], it is not eliminated, underscoring the importance of clinical monitoring and counseling [7]. In our study, it is unclear whether HIV+ women were instructed not to breastfeed or if they did not receive proper lactation support. Changes in clinical guidance and practice over the study period may have influenced breastfeeding patterns and therefore may contribute to the observed differences between groups.

The breastfeeding-stratified analyses are exploratory analyses, and the limitations imposed by the marked imbalance in exposure patterns are acknowledged. It should be mentioned that breastfeeding duration differed between HUU and HEU groups. Among HUU infants, the largest proportion (37%) were breastfed for 1–13 weeks after birth compared with 61.5% of HEU infants. However, the difference should be interpreted cautiously, as breastfeeding duration may be influenced by multiple maternal, infant, and social factors, including adherence to breastfeeding recommendations or clinical counseling. These factors were not evaluated in the present analysis, and further investigation is needed to determine the reasons for the observed differences.

Previous studies have demonstrated that HEU children may have altered immune regulation, including differences in cytokine production, innate immune responses, vaccine responses, and inflammatory pathways compared with HIV-unexposed, uninfected (HUU) children [1,9]. In addition, maternal HIV infection, maternal immune activation, or antiretroviral exposure during pregnancy may shape fetal immune development before birth, suggesting that prenatal exposures may have a bigger influence on long-term immune outcomes than postnatal feeding practices alone [11]. Breast milk composition among WLHIV may also differ from that of women without HIV, potentially affecting the extent to which breastfeeding provides immunologic benefits for HEU infants [16].

While Fourman et al. identified a higher prevalence of reactive airway disease (RAD) among HEU adolescents and young adults compared with HUU controls, their study did not include early-life manifestations of atopy or asthma and did not consistently document exposure to breast milk for their cohort [17]. In contrast, this current study evaluated asthma/atopic outcomes in relation to breastfeeding history among HEU and HUU children. Although breastfeeding remains an important source of nutrition and immune support, our study identifies an observed association between HIV exposure status and documented asthma/atopic outcomes among HEU children but cannot demonstrate that this is caused by HIV exposure itself.

This study has several limitations that should be considered: it is a retrospective study, has a relatively small sample size, and relies on documented clinical diagnoses. In addition, the limited/small number of breastfed HEU children reduced the ability to assess potential effects of breastfeeding duration. We also acknowledge the small number of HUU/breast milk not-exposed participants and the resulting uncertainty of estimates. There is an imbalance in breastfeeding status between the HEU and HUU groups. The small size of the breastfeeding-stratified subgroups, particularly the breast milk non-exposed HUU subgroup, resulted in limited statistical power and wide-confidence intervals. The absence of statistical significance should not be interpreted as evidence that breastfeeding does not affect asthma or atopic outcomes; it should be interpreted as insufficient evidence to establish an association, rather than evidence that no association exists. Despite these limitations, this study contributes important information regarding the role of breast milk exposure in HEU children.

## 5. Conclusions

As infant recommendations for WLHIV continue to evolve, understanding the long-term immunologic consequences of breastfeeding among HEU children will be essential for optimizing clinical care and infant-feeding practices among families affected by HIV.

## Figures and Tables

**Table 1:** Demographic and clinical characteristics of HIV-exposed uninfected (HEU) and HIV-unexposed uninfected (HUU) children.

Characteristic	Category	HEU (N = 67)	HUU (N = 58)
		N (%)	N (%)
**Age (years)**	<1	8 (11.94)	6 (10.34)
	1–4	39 (58.21)	39 (67.24)
	5–8	5 (7.46)	6 (10.34)
	9–12	7 (10.45)	5 (8.62)
	13–16	5 (7.46)	2 (3.44)
	17–21	3 (4.48)	0 (0)
**Gender**	Female	36 (53.73)	29 (50)
	Male	31 (46.27)	29 (50)
**Race/ethnicity**	Black, Non-Hispanic	61 (91.04)	52 (89.66)
	Black, Hispanic	2 (2.99)	1 (1.72)
	White, Non-Hispanic	2 (2.99)	2 (3.45)
	White, Hispanic	2 (2.99)	2 (3.45)
	Other	0 (0)	1 (1.72)
**Feeding**	Exposed to breast milk	13 (19.12)	54 (93.1)
	Not exposed to breast milk	54 (80.6)	4 (6.9)
**Atopic Outcomes**	Asthma	10 (14.93)	5 (8.62)
	Allergic rhinitis	9 (13.43)	1 (1.72)
	Atopic dermatitis	22 (32.84)	12 (20.69)
**Breastfeeding Duration (weeks)**	<1	1 (1.49)	0 (0)
	1–13	8 (61.5)	20 (37.0)
	14–26	2 (2.98)	9 (15.52)
	27–39	1 (1.49)	8 (13.79)
	40–52	0 (0)	5 (8.62)
	53–65	0 (0)	4 (6.90)
	66–78	0 (0)	4 (6.90)
	79–91	1 (1.49)	1 (1.72)
	92–104	0 (0)	2 (3.45)
	104<	0 (0)	1 (1.72)
**Allergy Family History**	Seasonal	7 (10.45)	3 (5.17)
	Eczema	6 (8.96)	3 (5.17)
	Asthma	15 (22.39)	18 (31.03)
	Food	5 (7.46)	3 (5.17)
	Drug	5 (7.46)	1 (1.72)

Mean age HEU (years): 5 ± 5; Mean Age HUU (years): 4 ± 4. Study period: 2005–2026.

**Table 2:** Association between HIV exposure status and asthma/atopy according to feeding status.

Feeding Status	HEU With Asthma/Atopy, n/N (%)	HUU with Asthma/Atopy, n/N (%)	Odds Ratio (HEU vs. HUU)	95% CI	Fisher’s Exact *p*-Value
**Breastfeeding-exposed**	6/13 (46.2)	18/54 (33.3)	1.71	0.54–5.46	0.31
**Non-breastfeeding-exposed**	33/54 (61.1)	1/4 (25.0)	4.71	0.46–48.43	0.19
**All children**	39/67 (58.2)	19/58 (32.8)	2.86	1.29–6.33	0.006

HEU: HIV-exposed uninfected; HUU: HIV-unexposed uninfected; OR: odds ratio; CI: confidence interval. Values are presented as n/N (%). Odds ratios compare HEU with HUU children, with HUU as the reference group. Two-sided Fisher’s exact tests were used because of the small N number of subjects. 95% confidence intervals are reported for the odds ratios.

## Data Availability

The data supporting the findings of this study are available from the corresponding author upon reasonable request.

## Abbreviations

The following abbreviations are used in this manuscript:

HIV	Human immunodeficiency virus
WLHIV	Women living with HIV
HEU	HIV-exposed, uninfected
HUU	HIV-unexposed, uninfected
